# Normative Data for a Multi-Domain Concussion Assessment in the Female Community Sport of Ladies Gaelic Football

**DOI:** 10.3390/sports13110405

**Published:** 2025-11-12

**Authors:** Róisín Leahy, Keith D. Rochfort, Enda Whyte, Anthony P. Kontos, Michael W. Collins, Siobhán O’Connor

**Affiliations:** 1 School of Health and Human Performance, Dublin City University, D09 YH9P Dublin, Ireland; 2 School of Biotechnology, Dublin City University, D09 Y074 Dublin, Ireland; 3 Life Sciences Institute, Dublin City University, D09 Y074 Dublin, Ireland; 4 Department of Orthopedic Surgery, University of Pittsburgh, Pittsburgh, PA 15260, USA

**Keywords:** athletic injuries, brain injury, diagnosis, rehabilitation, post-concussion syndrome

## Abstract

Due to the highly individualised presentation of sport-related concussion (SRC), multi-domain assessments examining cognitive, migraine, vestibular, ocular, mood, sleep, and neck-related function have been suggested to assist clinicians with diagnosis, management, and rehabilitation. Normative data on such assessments for female, community players from countries outside the U.S. are needed. This study aimed to (i) describe normative data from community-level Ladies Gaelic Football players using a multi-domain assessment, and (ii) compare findings between adolescent and adult players. A total of 138 LGF players without SRC (101 adults, 37 adolescents) completed a multi-domain SRC assessment including Sport Concussion Assessment Tool 5th Edition, Concussion Clinical Profiles Screening, Vestibular/Ocular Motor Screening (VOMS), Immediate Post-Concussion Assessment and Cognitive Testing (ImPACT^®^), Patient Health Questionnaire-9, Generalised Anxiety Disorder-7, Pittsburgh Sleep Quality Index, Migraine Disability Assessment, and Neck Bournemouth Questionnaire, and neck dynamometry. Normative data were summarised using descriptive statistics, while differences in test scores between adolescents and adults were examined using parametric or non-parametric tests. While adolescents and adults scored similarly on most measures, adolescents scored worse on ImPACT^®^ visual–motor speed (d = 0.09) and reaction time (r = 0.52), SCAT5 concentration (V = 0.38), total modified Balance Error Scoring System (r = 0.42), and CP Screen vestibular profile (r = 0.38) (*p* < 0.05). This is the first study to describe and compare normative data for multidomain SRC assessments in adolescent and adult female, community athletes. Differences in some tests between adolescents and adults highlight the need for demographic-specific normative data when interpreting post-SRC assessment results.

## 1. Introduction

Sport-related concussion (SRC) is a head trauma-induced condition with a highly varied symptomatology, including headaches, cognitive deficits, sensory disturbances, impaired balance and coordination, mood changes, and sleeping problems [1]. These varying symptoms may reflect different SRC subtypes, which may require targeted screening tools and rehabilitation [2,3]. Several conceptual models of SRC subtypes have been developed [2,3]. The clinical profiles model is a commonly applied model which hypothesises that SRCs initially present with general cognitive, fatigue and migraine-type symptoms (i.e., headaches accompanied by photo- or phonophobia), before developing along cognitive, migraine, mood, vestibular, or ocular/visual disturbance trajectories. These trajectories may overlap and be modified by sleep and neck-related factors [3]. Psychosocial factors may also interact with cognitive outcomes and should be considered during concussion assessment [4]. The clinical profiles model describes the risk factors, assessment findings, and outcomes of each trajectory, acknowledging the heterogeneity of SRC and the need for an individualised approach to diagnosis and rehabilitation. An individualised approach to SRC management may be particularly relevant for female athletes, who are more susceptible to sustaining concussions and developing persistent symptoms [5]. While SRC symptoms typically resolve without treatment within one month, a minority of players experience persistent symptoms [6]. In females this may reflect to a combination of psychosocial, physiological, and hormonal factors [5].

SRC may be challenging to diagnose and manage due to its highly individual presentation, non-specific symptoms, and lack of objective clinical signs [1]. Therefore, multi-domain assessments, incorporating tests for cognitive, migraine, vestibular, ocular, mood, sleep, and neck-related dysfunction, have been recommended [3,7]. Clinicians are often recommended to compare post-concussion test scores to pre-injury baseline scores when assessing players with a suspected SRC and when managing the RTP process [1,8]. However, collecting baseline data is resource-intensive and may be challenging to implement in community sport due to financial limitations. Comparing post-concussion test scores to normative data is an alternative approach which has been shown to have adequate diagnostic accuracy across a range of assessments [9,10]. Normative data describe the range of a test score in a healthy population with similar demographic characteristics [11]. While normative data exist for SRC tests, such as the Sport Concussion Assessment Tool (SCAT5) [12,13], Immediate Post-Concussion Assessment and Cognitive Testing (ImPACT^®^) [14,15,16], and Vestibular/Ocular Motor Screening (VOMS) [17], these datasets mainly feature North American youth [14,15,17], collegiate [16] or elite athletes [12], or international elite male athletes [13]. As baseline scores may be influenced by factors such as age and gender [11] it is important that normative data are drawn from a suitable population. Normative data are also lacking on assessments of domains including migraine/headache, anxiety/mood, neck, and sleep, such as those in the Concussion Clinical Profiles Screening (CP Screen) tool [18].

Ladies Gaelic Football (LGF) is one of the most popular team sports among females in Ireland. It is an amateur community sport, predominantly played between local teams and featuring players of varying skill levels [19]. However, there is also an elite, amateur division involving competitions between regional (county) teams. LGF resembles Australian football and although it is officially a non-contact sport [20], head impacts may occur. Previous research has reported that 63% of LGF players who sustained an SRC were injured as result of contact with another player, while 26% collided with the ground, and the remainder sustained an impact from a ball, fence or goalpost [21]. Among collegiate LGF players it has been identified that 9% of injuries prospectively reported over two seasons were SRCs [22]. Similar to other community sports, there are limited medical and financial resources across the entire LGF playing population. Medical coverage at training and matches is often minimal and inconsistent [23], and many teams do not have a designated medical professional. This results in a limited capacity to carry out pre-injury baseline testing of individual players. Normative SRC assessment data from female, community athletes are therefore urgently needed and would benefit both LGF and female community sport internationally. The main aim of this study was to provide normative data on a multi-domain SRC assessment for LGF players. A secondary aim was to examine differences in baseline test outcomes between adolescent and adult players.

## 2. Materials and Methods

### 2.1. Participants

The university Research Ethics Committee granted approval to carry out this study. This study complies with the guidelines for conducting research involving human participants as described in the Declaration of Helsinki [24]. Before being assessed, adult participants provided written informed consent. Adolescent participants provided written informed assent while their parents provided written informed consent. Participants were eligible for inclusion in this study if they were at least 13 years old and were currently playing with an LGF team. We excluded players who reported either being diagnosed with a concussion by a medical professional or sustaining a suspected concussion for which they did not seek medical attention in the 90 days preceding data collection. Participants reported concussions based on a standardised definition of concussion that was used in a previous study [23]. Concussions were classified as “diagnosed” if the participant had been diagnosed by a medical professional, and “suspected” if they had not been diagnosed by a medical professional.

Local collegiate and club LGF teams were recruited using convenience sampling. Local teams were targeted to increase participation in this study as data collection involved a visit to the university laboratory. The principal investigator contacted the coaches of four collegiate LGF teams, who agreed to participate in this study. Local club teams were also identified and contacted by the Ladies Gaelic Football Association (LGFA), who partially funded this project. The principal investigator visited all teams who were interested in participating in this study and explained the aims, requirements, and benefits of participation to the players or their parents. Individual players or the parents of adolescent players who wished to participate in this study then provided contact details. Participants were recruited from four collegiate teams, two adult club teams, and two underage club teams between June 2019 and February 2020.

### 2.2. Design

Measures were selected to form a multi-domain assessment based on the clinical profiles model. A comprehensive literature review was carried out to identify existing SRC assessments, as well as general patient reported outcome measures for mood, sleep, neck pain and migraine. The validity and test–retest reliability of all tests were reviewed and the most robust test selected was to assess each domain. Detailed descriptions, validity and reliability of included tests are reported in Appendix A. The following tests were selected: ImPACT^®^, a computerised neurocognitive assessment (cognitive) [25]; the VOMS, which assesses symptom provocation in response to eye and head movements (vestibular, ocular) [26]; neck dynamometry, which involves examining isometric neck strength using a hand-held dynamometer (HHD) (neck) [27,28]; the CP Screen, a symptom report tool based on the clinical profiles model [18]; the Patient Health Questionnaire 9 (PHQ-9) [29] and Generalised Anxiety Disorder-7 (GAD-7), self-report mood and anxiety instruments [30]; the Pittsburgh Sleep Quality Index (PSQI), a tool measuring self-reported sleep quality [31]; the Migraine Disability Assessment (MIDAS), which assesses self-reported migraine severity and dysfunction [32]; and the Neck Bournemouth Questionnaire (NBQ), a self-reported assessment of multiple domains of neck pain [33]. The SCAT5 [34,35], a composite sideline assessment consisting of symptom reporting, cognitive tests, and a neurological and balance assessment, was also included as it is considered the “gold-standard” tool for SRC.

### 2.3. Procedure

The full assessment took approximately 90 min per participant. Most (87.5%, *n* = 133) participants completed the assessment over two days, separated by a median of 9 days (IQR: 7). Demographic and sport-specific characteristics, relevant medical history, and concussion history were documented using self-report questionnaires. Height (cm) and mass (kg) measurements were recorded using a combined stadiometer and weighing scales (Electric Column Scale, seca GmbH & Co. KG, Hamburg, Germany).

The tests simultaneously to groups of up to twelve participants in a university computer lab or tutorial room. Participants completed ImPACT^®^ using a desktop computer or a laptop connected to an external mouse. Paper versions of questionnaires were administered. These tests were supervised by the principal investigator, a research staff member, or a research intern in the department. Participants were individually assessed for the SCAT5, VOMS, and neck dynamometry. The principal investigator, a qualified athletic therapist, and a trained research intern performed these tests in a biomechanics lab or an unused club changing room. The principal investigator carried out the SCAT5 neurological tests and mBESS, VOMS, and neck dynamometry, while the assistant carried out the SCAT5 Graded Symptom Checklist (GSC) and Standardised Assessment of Concussion (SAC). As a station format was used for testing, test order was not standardised. Considering the low number of testers and limited resources, a station format was considered the most efficient method of completing data collection. Assessments were carried out between August 2019 and March 2020. The tests outlined in Section 2.2 were carried out using methodology described in the literature (see Appendix A) [18,25,26,27,28,29,30,31,32,33,34,35]. ImPACT^®^ (version 3.11.1, “baseline” option) and questionnaires were administered.

### 2.4. Statistical Analysis

Data cleaning and analysis were carried out in RStudio (version 4.1.0, RStudio, Boston, MA, USA). Missing data accounted for 1.2% of the data (0.7–14.5% of values for individual variables). When missing data were caused by unit non-responses (e.g., participants failing to return for the second part of the assessment, equipment or software malfunctions, or invalid ImPACT^®^ results) available case analysis was used. Item non-responses (e.g., tester errors or failure of participants to answer all questionnaire items) were imputed using median imputation. Following imputation, we calculated test scores for all outcomes. We also created categorical variables for tests with recommended clinical cut-off thresholds and classification grades.

We presented demographic details, sporting history, and relevant medical and concussion history for total, adolescent, and adult participants. Players were characterised as elite if they reported playing with a county team at the time of testing. All test outcomes were initially plotted and visually examined. Continuous outcomes were assessed for normality using the Shapiro test and Q-Q plots, and for homogeneity of variance using Levene’s test. Descriptive statistics for test outcomes were presented for all participants and by age group. The proportion of participants meeting clinical cut-off thresholds and each classification grade for the VOMS, PHQ-9, GAD-7, PSQI and MIDAS were presented for all participants and by age group using valid percentages. The following clinical cut-off thresholds were used: ≥10 (PHQ-9) [29], ≥10 (GAD-7) [30], and ≥5 (PSQI) [31]. The PHQ-9 and GAD-7 were further classified by severity (PHQ-9: 0–4 (none), 5–9 (mild), 10–14 (moderate), 15–19 (moderately severe), 20–27 (severe) [29]; GAD-7: 0–4 (none), 5–9 (mild), 10–14 (moderate), 15–21 (severe) [30]. The MIDAS was classified into four grades: scores of 0–5 (GI), 6–10 (GII), 11–20 (GIII), and ≥21 (GIV) [36]. No clinical cut-off thresholds have been suggested for the NBQ.

Between-group analyses were performed to examine differences in test outcomes between adolescent and adult participants. Differences in normally distributed continuous outcomes were assessed using two-tailed independent *t*-tests with Welch corrections to account for unequal sample sizes and variances. Wilcoxon rank-sum tests were selected to examine differences in continuous outcomes when the assumption of normality was not met. Between-group differences in categorical outcomes were assessed using two-tailed Chi-square tests of independence if fewer than 20% of expected cell frequencies were less than five; otherwise, we used Fisher exact tests. Effect sizes were expressed as Glass’s delta for *t*-tests (small: 0.2, moderate: 0.5, large: 0.8) [37]. Glass rank biserial correlations for Wilcoxon rank-sum tests (with −1 indicating that all values in the second group are greater than all values in the first group, and +1 indicating that all values in the first group are greater than all values in the second group) [38], and Cramer’s V for Chi-square and Fisher exact tests (small: 0.1; moderate: 0.3; large: 0.5) [39]. An a priori alpha level of 0.05 was selected. Holm–Bonferroni procedures were applied to analyses to minimise the family-wise error rate.

## 3. Results

### 3.1. Demographic Characteristics

One hundred and fifty-two participants completed the initial assessment; however, 14 participants were removed from the dataset due to a suspected or diagnosed concussion within the previous three months (*n* = 8) or missing information about concussion history (*n* = 6). The final sample contained 138 (101 adult, 37 adolescent) participants. Participants had a median age of 19 (IQR: 3.0) and approximately 30% played at an elite level. One-fifth reported a previous diagnosis of concussion by a medical professional. Relevant participant characteristics are presented in Table 1.

### 3.2. Concussion Assessment

#### 3.2.1. Sport Concussion Assessment Tool 5th Edition

Participants scored a median of four (out of 22) for the Total Symptom Score (TSS) and five (out of 132) for Symptom Severity Score (SSS) (see Table 2). Most (92.5%, *n* = 124) participants reported experiencing some concussion-like symptoms when asked how they “typically feel” on the SCAT5 GSC; fatigue (66.4%, *n* = 89), difficulty concentrating (47.8%, *n* = 64), and feeling nervous or anxious (42.5%, *n* = 57) were most frequent (see Appendix B).

One-fifth (20.1%) of participants failed to correctly answer all five SCAT5 orientation questions. A minority (7.5%) of participants were unable to correctly name all of the months backwards in the SCAT5 concentration component. Participants completed the finger-to-nose test in a mean time of 4.4 (±0.9) seconds, and the TG test in a median time of 25.2 s. Two (1.5%) participants were unable to complete the TG test. The median mBESS score was 5, with participants scoring most poorly in the single-leg stance condition (median: 3.0). Adult participants scored significantly higher on the SAC “digits backwards” (*p* = 0.037, V = 0.36) and total concentration (*p* = 0.015, V = 0.38) tests, with moderate effect sizes, and significantly lower on the mBESS tandem gait stance (*p* = 0.006, r = 0.42) and mBESS total (*p* = 0.009, r = 0.42) scores. Results are reported in detail in Table 2.

#### 3.2.2. Clinical Profiles Screen

Participants reported a median CP Screen total score of six. Highest median scores were reported for the cognitive profile [1], followed by the ocular [1] and anxiety/mood [1] profiles and sleep modifier [1]. The median score for each of the remaining profiles was zero (see Table 3). Adolescents scored significantly higher on the vestibular profile than adults (*p* = 0.007; r = 0.38). No other significant differences were observed between age groups (*p* > 0.05).

#### 3.2.3. Vestibular/Ocular Motor Screening

The median change score was zero for all individual VOMS items, and one for the VOMS Total Symptom Score (see Table 4 and Appendix C). Individual VOMS item scores or total symptom scores did not differ between adolescents and adults (*p* > 0.05).

#### 3.2.4. Immediate Post-Concussion Assessment and Cognitive Testing

Median ImPACT^®^ composite scores of 87.5 (verbal memory), 73.0 (visual memory), 37.4 (visual motor speed), and 0.6 (reaction time) were recorded for all participants (see Table 4). Adolescent participants had significantly lower visual motor speed (*p* < 0.001, d = 0.09) and slower reaction time scores (*p* < 0.001, r = 0.52) than adult participants.

#### 3.2.5. Mental Health

The median scores were 3.0 for the PHQ-9 and 1.5 for the GAD-7 [see Table 5]. Most participants were graded as “none” (72.1%, *n* = 98) or “mild” (21.3%, *n* = 29) on the PHQ-9, with 6.6% (*n* = 9) meeting the recommended clinical cut-off threshold for depression (score of ≥10). Over three-quarters (75.7, *n* = 103) of participants were classified “none” on the GAD-7, and 10.3% (*n* = 14) met the recommended clinical cut-off threshold for anxiety (score of ≥10). There were no significant differences in any PHQ-9 or GAD-7 outcomes between groups (*p* > 0.05).

The median global PSQI score for all participants was 5, with subjective sleep quality (1), sleep latency (1), sleep disturbance (1), and daytime dysfunction (1) components demonstrating high median scores. Over half (56.3%, *n* = 76) of participants were classified as having poor quality sleep (global score of ≥5). PSQI outcomes did not differ based on age group (*p* > 0.05).

#### 3.2.6. Neck

Participants recorded mean neck dynamometry values of 1.2 (±0.3) N/kg body mass for flexion, 1.3 (±0.3) N/kg for extension, 1.0 (±0.2) N/kg for dominant-side lateral flexion, 1.0 (±0.2) N/kg for non-dominant side lateral flexion, and 0.7 (±0.2) N/kg for non-dominant side rotation. The median value for dominant side rotation was 0.7 (IQR: 0.6–0.8) (see Table 6). Normalised neck strength did not differ based on age (*p* > 0.05). The median NBQ score was 3.0 (see Table 6). Between-group analyses did not find any statistically significant differences in any NBQ (*p* > 0.05).

#### 3.2.7. Migraine

Participants reported a median number of 0 days disability due to migraine over the previous three months on the MIDAS. Most participants (83.8%, *n* = 114) met the criteria for a MIDAS grade I (little or no disability). MIDAS scores did not show any significant differences based on age (*p* > 0.05) (see Table 5).

## 4. Discussion

This is the first study to describe normative data on a multi-domain SRC assessment in both adult and adolescent female, community sport athletes. We presented normative data on multiple measures, including the SCAT5, CP Screen, VOMS, ImPACT^®^, PHQ-9, GAD-7, PSQI, neck dynamometry, NBQ, and MIDAS. While most measures were similar between age groups, adolescents had worse ImPACT^®^ visual motor speed and reaction time, SCAT5 concentration, mBESS, and CP Screen vestibular scores.

Participants in our study reported a median of four symptoms on the SCAT5 GSC. The most common symptoms were fatigue (66.4%), difficulty concentrating (47.8%), and feeling nervous or anxious (42.5%). Baseline reporting of concussion-like symptoms is common in female athletes [40]. Concussion symptoms are non-specific and may present in players without concussion due to underlying medical conditions (e.g., migraine [41] or mood [42] disorders) or hormonal fluctuations [43]. However, participants in this study reported considerably more symptoms than previously reported by female collegiate and professional athletes (TSS: 0–1, SSS: 0–2) [12,34,44]. Due to low rates of relevant medical conditions (e.g., LDs, ADHD) among participants in our sample, we were unable to examine baseline symptom differences between players with and without these conditions. While participants with these conditions represented a small percentage (0.0–15.4%) of the overall sample, high TSSs and SSSs among these participants may nevertheless have skewed GSC scores. Clinicians should consider demographic and medical history characteristics that may affect baseline symptoms when managing RTP following an SRC. We recommend that future studies expand on our research and present normative data for female, community players with a history of relevant medical conditions.

We reported similar median scores for the SAC as previous studies of adolescent [45,46], collegiate [47], and elite adult [12] female athletes: five for orientation, 20 for immediate recall, four for concentration, and six for delayed recall. However, adolescent participants scored significantly lower than adult participants on the digits backwards test, contributing to significantly lower concentration scores with a moderate effect size. Few previous studies have examined the effect of age on SAC scores [45,48]. One study comparing older (aged 16–19) and younger (aged 13–15) female adolescents reported lower mean concentration scores (3.0 vs. 3.6) in the younger athletes [48]. Another study found no age effects in high school players’ concentrations scores, however 75% of participants were male and separate analyses for male and female players were not performed [45]. Differences in concentration abilities between adolescents and adults may reflect natural neurocognitive development processes [49]. As neurodevelopment rates differ between males and females [50], it is essential that clinicians consult normative data from the appropriate population when assessing players with the SAC.

Our participants mBESS scores (median of 5/10) were higher than the median scores of 2–3 that have been reported in female youth [51], high school [51], collegiate [34,51], and elite rugby [12] players. Relatively poor static postural stability in our participants may have been due to the community nature of LGF and the mixture of skill levels across participants. However, previous studies did not provide detailed information about how the mBESS was carried out. As the SCAT5 is intended for sideline usage, variation in some aspects of the protocol is permitted [35]. We assessed participants standing barefooted on a firm surface; for the single-leg stance participants stood on their non-dominant leg, while for the tandem gait stance they placed their non-dominant foot behind their dominant foot. Clinicians consulting our normative data should follow the same protocol. In addition, adolescent players in this study had worse total and tandem gait stance scores than adult players. Improved mBESS scores have been reported with increasing age in adolescents [51,52]. The sensorimotor systems, which are responsible for balance, develop throughout adolescence [53], possibly explaining the differences in mBESS scores between adolescents and adults. Clinicians working with female, community sport athletes should ensure that they consult age-specific normative data when carrying out SRC balance assessments.

Participants took a median of 4.4 s to complete the finger-to-nose (FTN) test, which is slower than has previously been reported in healthy adults (mean of 2.9–3.0 s) [54]. While the FTN test is incorporated into the SCAT5, timing the test is not mandatory and we performed it as additional measure. Although there is minimal data available on time to complete the FTN test in athletes, our participants were slower to complete the test than may be expected. This may have been due to the fact that they completed it as part of the multidomain assessment and not as a standalone test.

While the CP Screen has been used to examine post-SRC symptoms in adolescents and adults [55], normative data have not been previously collected in any population. Our participants demonstrated the highest median scores on the cognitive (0.3), ocular (0.2), and anxiety/mood profiles (0.2). These profiles include non-specific symptoms (e.g., memory difficulties, headaches, eye strain, rumination, and feeling anxious), which may be explained by psychological stress [42], environmental factors [56] or underlying medical conditions [41,57] in healthy players. Our results support existing literature reporting that female players frequently experience mild concussion-like symptoms even when uninjured [25,40,58]. It is important that clinical decisions are made taking a range of factors and assessment findings into consideration as it may not be possible for some female athletes to be completely symptom-free.

We reported moderately higher CP Screen vestibular scores for adolescents than adults. Vestibular symptoms include dizziness, motion sickness, and discomfort in busy environments [18]. There are little data available on the prevalence of vestibular dysfunction in adolescents [59] and none comparing adolescents and adults. It is possible that adolescents experience greater vestibular dysfunction relative to adults due to ongoing development of the vestibular system throughout adolescence [53]. Alternatively, age differences in vestibular scores may have been due to a confounding factor, such as a history of migraine or motion sickness, which have been associated with vestibular dysfunction [59,60]. Three-times as many adolescents (18.9%, *n* = 7) as adults (6.1%, *n* = 6) self-reported a history of migraine, however our sample size was insufficient to analyse differences in outcomes based on medical history. Furthermore, we did not assess participants for motion sickness susceptibility. Additional research is required to understand differences in self-reported vestibular function in adolescent players compared with adult players before recommendations can be made to clinicians.

We reported median change scores of zero for all VOMS items, as well as a median NPC distance of zero centimetres, with no differences found based on age. Females are more likely to suffer from migraine [61], motion sickness [62], and anxiety [63] than males, all of which are associated with vestibular dysfunction [64,65]. Due to the small proportion of participants who had been diagnosed with migraine (*n* = 13) or anxiety (*n* = 2), we were unable to examine differences in VOMS results based on medical history. However, these conditions may be underdiagnosed and there is a need for more extensive normative data on the VOMS in female athletes that takes relevant medical history into account. To date, several clinical cut-off points for the VOMS have been suggested [26,66], however there is a lack of consensus on the most useful cut-off points. Further research on developing clinical cut-off points, particularly in female athletes, is needed.

Previous studies have reported broad ranges of mean ImPACT^®^ composite scores in adolescent and adult female players: 82.5–99.1 (verbal memory), 70.8–80.6 (visual memory), 29.0–44.0 (visual motor speed), and 0.55–0.62 (reaction time) [15,16,25,67,68]. Similarly, we reported overall median ImPACT^®^ scores of 87.5 for verbal memory, 73.0 for visual memory, 37.4 for visual motor speed, and 0.6 s for reaction time. Demographic [25,67,68,69,70,71] and environmental [68] factors may influence neurocognitive assessment results. Therefore, establishing normative values is difficult. Clinicians consulting normative data should be aware of the vast array of factors that may influence results and ensure that they select appropriate normative data if available. Our normative data will greatly assist clinicians working with female, community sport athletes across a range of ages.

Adolescent participants had slower visual–motor speed and reaction time than adults. Improved visual motor speed [68,70] and reaction time [68,70] have been reported in older versus younger adolescents. Similarly, collegiate athletes demonstrate higher (45.8) visual motor speed scores compared with high school athletes (39.4) [71]. This may be because white matter development, which is associated with processing speed [72], continues throughout adolescence and into adulthood [49,72]. Our findings justify the need for separate norms for adolescent and adult players on ImPACT^®^ composite scores. However, adolescent participants ranged in age from 13 to 17 in our study. As considerable neurocognitive maturation takes places during this period [72], future research that stratifies adolescent participants into younger (e.g., 13–15) and older (e.g., 16–17) adolescents is needed.

Most SRC tests do not provide detailed information on subjective symptoms, such as low mood, sleep difficulties, neck pain and migraine. Incorporating measures of these symptoms into an SRC assessment may be useful in guiding rehabilitation in players who are suffering from persistent symptoms. Although normative data on these measures exist for various clinical populations, few studies have reported baseline scores in athletes [73,74,75,76,77,78,79]. As most of these measures examine symptoms over the previous week to three months, they may be most useful in managing the rehabilitation of players with persistent SRC symptoms.

Baseline data for the PHQ-9 and GAD-7 in athletes is very limited. Our participants were experiencing low levels of depression (median PHQ-9 of 3) and anxiety (median GAD-7 of 1.5) at the time of data collection. This is higher than the median PHQ-9 scores of zero on and GAD-7 scores of 0–1 that have been reported in male and female athletes [73,74]. This may be explained by the higher prevalence of depression and anxiety in females [80]. Depression and anxiety may be influenced by psychosocial [81] or behavioural factors [4], emphasising the need for population-specific normative data. Our study is the first to report normative data for the PHQ-9 and GAD-7 in female, community sport athletes and will assist clinicians in managing players with persistent post-SRC mood impairment. The proportion of participants who met the clinical cut-off threshold for depression (6.6%) and anxiety (14%) in our study was considerably higher than the proportion who reported previously being diagnosed with a mood (2.2%) or anxiety (1.5%) disorder. Mood and anxiety disorders are thought to be underdiagnosed, and symptom reports may provide a more accurate measure of a player’s mental health than medical history. There is a need for increased awareness of mental health conditions among female, community athletes as pre-existing mood disorders may increase the risk of mood-related symptoms post-SRC [82]. As well as guiding the management of players with persistent mood symptoms, the PHQ-9 and GAD-7 may also be useful as baseline screening tools to help clinicians identify players at risk of post-SRC mood disturbances and develop suitable interventions.

This is the first study to present normative data on neck strength in female athletes, regardless of age, sport, or playing level. Cervical spine injuries may co-occur with SRC, resulting in headaches, neck pain, and muscle weakness [2,83]. Incorporating neck dynamometry into SRC assessments may assist clinicians in managing the RTP and rehabilitation processes. Surprisingly, no differences were observed in normalised neck strength based on age group. Adults typically demonstrate increased muscle strength relative to adolescents [84]. It is possible that normalising force to body mass concealed strength differences between adolescents and adults. Clinicians working with female, community athletes may find this normative data useful when managing patients presenting with cervical spine symptoms alongside an SRC.

Participants reported low baseline levels of neck pain (median NBQ of three) and disability due to migraine (83.8% reported a MIDAS grade I), with no differences observed between adults and adolescents. The NBQ and MIDAS were originally developed for use in clinical populations and our study is the first to use them in athletes. Neck injuries may occur alongside SRCs due to a shared mechanism of injury [85]. As symptoms of neck injuries and SRC may overlap (e.g., dizziness, poor balance and visual problems) [1,86], assessing neck function is an important part of the SRC assessment. Our data shows that self-reported neck impairment is uncommon in LGF players without SRC. Similarly, while players with SRC often present with post-traumatic migraine, migraine appears to be rare in those without SRC [87]. The NBQ and MIDAS may be useful in assisting clinicians who are managing players with persistent neck or migraine symptoms following an SRC. In addition, as pre-existing migraine may be a risk factor for a prolonged recovery from SRC [88], incorporating the MIDAS into baseline SRC assessments may be useful in identifying players who may be at an increased risk of SRC complications.

Over half (56.3%) of participants experienced poor sleep quality (global score ≥ 5). High rates of poor sleep quality have also been reported in high school, collegiate and elite athletes, with 40.4–67.0% scoring ≥ 5 on the PSQI in previous studies [75,78,79,89]. Participants scored worst on the subjective sleep quality, sleep latency, sleep disturbance, and daytime dysfunction components of the PSQI, similar to findings from elite female athletes [75]. This suggests that poor sleep quality in our participants may have been due to factors such as difficulty falling asleep and interruptions during sleep, as opposed to not sleeping for long enough. Our study is the first to present normative PSQI data for female, community athletes. LGF players often train and compete at a high level in addition to attending school, college or work full-time, which may have contributed to poor sleep among our participants. Sleeping difficulties commonly occur following an SRC [12,40,90]. In addition, pre-existing sleeping difficulties have been associated with a greater symptom burden and neurocognitive impairments following an SRC [91]. The PSQI may assist clinicians in managing players with persistent sleeping difficulties following an SRC. In addition, it may be useful in identifying players with pre-existing sleeping difficulties if used as part of a baseline assessment.

### 4.1. Limitations

This study has several limitations. Firstly, the development of the SRC assessment was guided by the clinical profiles model of concussion [3], which was developed based on clinical expertise and lacks empirical validity. It is possible that the suggested clinical profiles do not represent distinct entities. Secondly, the study sample may not represent a true baseline group, with one-fifth of participants reporting a previous diagnosed concussion. While we excluded participants who had sustained a suspected or diagnosed concussion in the 90 days prior to data collection, a small proportion of participants who had suffered from concussions beyond this timeframe may still have experienced lingering, long-term effects at the time of testing. This may have biassed our findings by negatively impacting neurocognitive and balance assessments and increasing the number of symptoms reported. Similarly, using convenience sampling may have resulted in a self-selection bias, potentially resulting in participants with a history of SRC being overrepresented in the sample. Thirdly, as our main aim was to present normative data a formal sample size calculation was not carried out. There were a relatively small number of adolescent participants, meaning that this study may have been underpowered to detect small effect sizes between groups. This imbalance in group numbers occurred as data collection was prematurely stopped due to the COVID-19 pandemic. Fourthly, variations in the testing protocol (e.g., participants completing the assessment across two days, lack of standardised test order, and differences in testing location) may have resulted in some bias in the results. Fifthly, a low proportion of participants reported a history of relevant medical conditions, preventing us from analysing differences in test outcomes between players with and without these conditions. Sixthly, this study includes LGF players only. While LGF is one of the most popular female sports in Ireland, the findings may not be generalisable to other female, community sports.

### 4.2. Directions for Future Research

Future research is needed to address the limitations of this study and improve the generalisability of the multi-domain SRC assessment. Including an additional control group consisting of athletes participating in a non-contact sport would reduce bias by removing the influence of previous SRCs on normative data. Future studies could also perform more in-depth research on differences in testing scores based on age group, competition level, SRC history, and relevant medical history.

## 5. Conclusions

This is the first study to describe and compare normative data for multi-domain SRC assessments in adolescent and adult female, community LGF athletes in Ireland. Our participants performed similarly to female athletes participating in other field sports on neurocognitive assessments and sleep quality questionnaires; however, they also reported more symptoms at baseline and had worse balance and motor control. In addition, adolescents in our study had significantly worse visual–motor speed, reaction time, concentration, balance and vestibular item scores than adults.

## 6. Practical Applications

This study provides population-specific normative data that clinicians can use to assist in the management of female, community athletes with suspected or confirmed SRC.

## Figures and Tables

**Table 1:** Participant demographic characteristics and medical history.

Demographic Characteristics	Total (*n* = 138)	Adolescent (*n* = 37)	Adult (*n* = 101)
**Level, % (*n*)**			
Elite	29.7 (41)	18.9 (7)	33.7 (34)
Non-elite	70.3 (97)	81.1 (30)	66.3 (67)
**Age in years, median (IQR)**	19.0 (3.0)	14.0 (1.0)	19.0 (2.0)
**Height in cm, median (IQR)**	166.5 (7.5)	166.6 (10.2)	166.5 (7.3)
Missing, % (*n*)	12.3 (17)	18.9 (7)	9.9 (10)
**Body mass in kg, median (IQR)**	65.3 (12.3)	59.4 (12.0)	66.6 (9.6)
Missing, % (*n*)	13.0 (18)	18.9 (7)	10.9 (11)
**Current level of education, % (*n*)**			
Completed undergraduate degree	8.8 (12)	0.0 (0)	12.1 (12)
Completed final school exams	66.2 (90)	8.1 (3)	87.9 (87)
Post-junior certificate	2.9 (4)	10.8 (4)	0.0 (0)
Pre-junior certificate	22.1 (30)	81.1 (30)	0.0 (0)
Other	0.0 (0)	0.0 (0)	0.0 (0)
Missing	1.4 (2)	0 (0)	2.0 (2)
**Number of years playing, median (IQR)**	12.0 (6.0)	8.00 (4.0)	13.0 (5.0)
Missing,	1.4 (2)	0 (0)	2.0 (2)
**Playing position, % (*n*)**			
Forward	47.8 (65)	45.9 (17)	48.5 (48)
Midfield	15.4 (21)	13.5 (5)	16.2 (16)
Back	31.6 (43)	32.4 (12)	31.3 (31)
Goalkeeper	3.7 (5)	5.4 (2)	3.0 (3)
Multiple	1.5 (2)	2.7 (1)	1.0 (1)
Missing	1.4 (2)	0 (0)	2.0 (2)
**Medical history**			
**Suspected or diagnosed concussion, % (*n*)**	31.2 (43)	21.6 (8)	34.7 (35)
**Diagnosed concussion, % (*n*)**	20.4 (28)	16.2 (6)	22.0 (22)
Missing	0.7 (1)	0 (0)	1.0 (1)
**Diagnosed headache, % (*n*)**	15.4 (21)	27.0 (10)	11.1 (11)
Missing	1.4 (2)	0 (0)	2.0 (2)
**Diagnosed migraine, % (*n*)**	9.6 (13)	18.9 (7)	6.1 (6)
Missing	1.4 (2)	0 (0)	2.0 (2)
**Diagnosed attention deficit hyperactivity disorder, % (*n*)**	0.7 (1)	0.0 (0)	1.0 (1)
Missing	2.9 (4)	2.7 (1)	3.0 (3)
**Diagnosed learning disorder, % (*n*)**	6.2 (8)	3.0 (1)	7.3 (7)
Missing	6.5 (9)	10.8 (4)	5.0 (5)
**Diagnosed mood disorder, % (*n*)**	2.2 (3)	2.8 (1)	2.0 (2)
Missing	2.2 (3)	2.7 (1)	2.0 (2)
**Diagnosed anxiety disorder, % (*n*)**	1.5 (2)	0.0 (0)	2.0 (2)
Missing	2.9 (4)	2.7 (1)	3.0 (3)
**Diagnosed sleep disorder, % (*n*)**	0.0 (0)	0.0 (0)	0.0 (0)
Missing	3.6 (5)	5.4 (2)	3.0 (3)

IQR = interquartile range.

**Table 2:** Descriptive statistics for Sport Concussion Assessment Tool 5th Edition.

Outcome	Age	N (m/)	Mean (SD)	Median (IQR)	Range	*p* -Value	Effect Size
**Graded Symptom Checklist**							
Total Symptom Score	Total	134 (4)	5.8 (4.7)	4.0 (2.0–9.0)	0.0–21.0	-	-
	Adolescent	37 (0)	7.8 (5.9)	7.0 (3.0–12.0)	0.0–21.0	0.831	0.27 (0.05–0.46) ^1^
	Adult	97 (4)	5.0 (3.9)	4.0 (2.0–8.0)	0.0–16.0	-	-
Symptom Severity Score	Total	134 (4)	8.8 (9.5)	5.0 (3.0–12.0)	0.0–60.0	-	-
	Adolescent	37 (0)	13.1 (13.4)	9.0 (3.0–18.0)	0.0–60.0	1.000	0.25 (0.04–0.45) ^1^
	Adult	97 (4)	7.2 (6.8)	4.0 (2.0–11.0)	0.0–32.0	-	-
**Standardised Assessment of Concussion**							
Orientation	Total	134 (4)	4.8 (0.4)	5.0 (5.0–5.0)	3.0–5.0	-	-
	Adolescent	37 (0)	4.7 (0.5)	5.0 (5.0–5.0)	3.0–5.0	1.000	0.15 (0.00–0.30) ^2^
	Adult	97 (4)	4.8 (0.4)	5.0 (5.0–5.0)	4.0–5.0	-	-
Immediate Memory	Total	134 (4)	19.8 (3.2)	20.0 (17.0–22.0)	11.0–27.0	-	-
	Adolescent	37 (0)	18.8 (2.9)	19.0 (17.0–21.0)	12.0–25.0	0.843	−0.20 (−0.55–0.14) ^3^
	Adult	97 (4)	20.2 (3.3)	21.0 (18.0–23.0)	11.0–27.0	-	-
Concentration	Total	134 (4)	4.0 (1.0)	4.0 (3.0–5.0)	1.0–5.0	-	-
	Adolescent	37 (0)	3.4 (1.0)	3.0 (3.0–4.0)	1.0–5.0	0.017	0.38 (0.17–0.53) ^2^
	Adult	97 (4)	4.2 (0.9)	4.0 (4.0–5.0)	2.0–5.0	-	-
Digits Backwards	Total	134 (4)	3.0 (0.9)	3.0 (2.0–4.0)	1.0–4.0	-	-
	Adolescent	37 (0)	2.5 (0.9)	2.0 (2.0–3.0)	1.0–4.0	0.041	0.36 (0.16–0.51) ^2^
	Adult	97 (4)	3.2 (0.9)	3.0 (3.0–4.0)	1.0–4.0	-	-
Months Backwards	Total	134 (4)	0.9 (0.3)	1.0 (1.0–1.0)	0.0–1.0	-	-
	Adolescent	37 (0)	0.9 (0.3)	1.0 (1.0–1.0)	0.0–1.0	1.000	0.08 (0.00–0.24) ^2^
	Adult	97 (4)	0.9 (0.2)	1.0 (1.0–1.0)	0.0–1.0	-	-
Delayed Recall	Total	133 (5)	6.1 (1.9)	6.0 (5.0–8.0)	0.0–10.0	-	-
	Adolescent	37 (0)	6.1 (1.6)	6.0 (5.0–7.0)	4.0–10.0	1.000	−0.01 (−0.23–0.20) ^1^
	Adult	96 (5)	6.0 (2.0)	6.0 (5.0–8.0)	0.0–10.0	-	-
**Neurological Screen**							
Finger-to-Nose Test, time	Total	134 (4)	4.4 (0.9)	4.4 (3.8–5.0)	2.6–7.1	-	-
	Adolescent	37 (0)	4.5 (0.8)	4.5 (3.9–5.0)	2.6–6.1	1.000	0.15 (−0.27–0.58) ^3^
	Adult	97 (4)	4.4 (1.0)	4.4 (3.6–5.1)	2.6–7.1	-	-
Tandem Gait, time	Total	132 (6)	26.4 (5.2)	25.2 (22.2–30.1)	16.2–42.0	-	-
	Adolescent	36 (1)	28.4 (5.8)	28.3 (23.6–32.8)	19.7–40.0	0.799	0.27 (0.06–0.46) ^1^
	Adult	96 (5)	25.6 (4.8)	25.0 (22.0–28.7)	16.2–42.0	-	-
**Modified Balance Error Scoring System Total**							
	Total	134 (4)	6.2 (4.7)	5.0 (3.0–8.0)	0.0–23.0	-	-
	Adolescent	37 (0)	8.7 (5.3)	7.0 (5.0–12.0)	1.0–23.0	0.010	0.42 (0.23–0.58) ^1^
	Adult	97 (4)	5.3 (4.2)	4.0 (2.0–7.0)	0.0–20.0	-	-
Double-Leg Stance	Total	134 (4)	0.0 (0.3)	0.0 (0.0–0.0)	0.0–3.0	-	-
	Adolescent	37 (0)	0.1 (0.5)	0.0 (0.0–0.0)	0.0–3.0	1.000	0.20 (0.00–0.35) ^2^
	Adult	97 (4)	0.0 (0.0)	0.0 (0.0–0.0)	0.0–0.0	-	-
Single-Leg Stance	Total	134 (4)	4.3 (3.2)	3.0 (2.0–6.0)	0.0–10.0	-	-
	Adolescent	37 (0)	5.6 (3.2)	5.0 (3.0–10.0)	1.0–10.0	0.139	0.34 (0.13–0.51) ^1^
	Adult	97 (4)	3.8 (3.1)	3.0 (2.0–6.0)	0.0–10.0	-	-
Tandem Gait Stance	Total	134 (4)	1.9 (2.4)	1.0 (0.0–2.0)	0.0–10.0	-	-
	Adolescent	37 (0)	3.0 (2.8)	2.0 (1.0–4.0)	0.0–10.0	0.007	0.42 (0.23–0.59) ^1^
	Adult	97 (4)	1.5 (2.2)	1.0 (0.0–2.0)	0.0–10.0	-	-

^1^ = Rank-biserial correlation; ^2^ = Cramer’s V; ^3^ = Glass’ delta; m/ = number of participants missing; IQR = Interquartile range; SD = Standard deviation.

**Table 3:** Descriptive statistics for Clinical Profile Screen outcomes.

Outcome	Demographic	N (m/)	Mean (SD)	Median (IQR)	Range	*p* -Value	Effect Size
**Clinical Profile Screen—Total Scores**							
Cognitive/Fatigue	Total	136 (2)	1.5 (1.4)	1.0 (0.0–2.0)	0.0–6.0	-	^-^
	Adolescent	37 (0)	1.9 (1.5)	2.0 (1.0–2.0)	0.0–6.0	1.000	0.22 (0.01–0.42) ^1^
	Adult	99 (2)	1.4 (1.3)	1.0 (0.0–2.0)	0.0–6.0	-	-
Ocular	Total	136 (2)	1.6 (2.0)	1.0 (0.0–3.0)	0.0–9.0	-	^-^
	Adolescent	37 (0)	2.1 (2.2)	2.0 (0.0–3.0)	0.0–8.0	1.000	0.19 (−0.03–0.39) ^1^
	Adult	99 (2)	1.4 (1.9)	1.0 (0.0–2.5)	0.0–9.0	-	-
Anxiety/Mood	Total	136 (2)	1.9 (2.2)	1.0 (0.0–3.0)	0.0–9.0	-	-
	Adolescent	37 (0)	2.8 (2.6)	2.0 (1.0–5.0)	0.0–9.0	0.130	0.33 (0.12–0.51) ^1^
	Adult	99 (2)	1.5 (2.0)	1.0 (0.0–2.0)	0.0–9.0	-	-
Migraine	Total	136 (2)	0.6 (1.1)	0.0 (0.0–1.0)	0.0–5.0	-	-
	Adolescent	37 (0)	0.9 (1.5)	0.0 (0.0–1.0)	0.0–5.0	1.000	0.17 (−0.05–0.37) ^1^
	Adult	99 (2)	0.4 (0.8)	0.0 (0.0–1.0)	0.0–4.0	-	-
Vestibular	Total	136 (2)	0.8 (1.3)	0.0 (0.0–1.0)	0.0–6.0	-	-
	Adolescent	37 (0)	1.6 (1.8)	1.0 (0.0–2.0)	0.0–6.0	0.007	0.38 (0.18–0.55) ^1^
	Adult	99 (2)	0.5 (0.9)	0.0 (0.0–1.0)	0.0–5.0	-	-
Sleep	Total	136 (2)	1.4 (1.6)	1.0 (0.0–2.0)	0.0–9.0	-	-
	Adolescent	37 (0)	1.5 (2.0)	1.0 (0.0–2.0)	0.0–9.0	1.000	−0.02 (−0.23–0.20) ^1^
	Adult	99 (2)	1.4 (1.4)	1.0 (0.0–2.0)	0.0–6.0	-	-
Neck	Total	136 (2)	0.3 (0.9)	0.0 (0.0–0.0)	0.0–6.0	-	-
	Adolescent	37 (0)	0.4 (0.8)	0.0 (0.0–0.0)	0.0–3.0	1.000	0.03 (−0.19–0.24) ^1^
	Adult	99 (2)	0.3 (0.9)	0.0 (0.0–0.0)	0.0–6.0	-	-
Total Score	Total	136 (2)	8.1 (7.7)	6.0 (3.0–10.0)	0.0–40.0	-	-
	Adolescent	37 (0)	11.3 (10.0)	8.0 (3.0–17.0)	0.0–40.0	1.000	0.26 (0.04–0.45) ^1^
	Adult	99 (2)	6.9 (6.3)	5.0 (3.0–9.0)	0.0–30.0	-	-
**Clinical Profile Screen—Average Scores**							
Cognitive/Fatigue	Total	136 (2)	0.5 (0.4)	0.3 (0.0–0.7)	0.0–2.0	-	-
	Adolescent	37 (0)	0.6 (0.5)	0.7 (0.3–0.7)	0.0–2.0	1.000	0.22 (0.01–0.42) ^1^
	Adult	99 (2)	0.4 (0.4)	0.3 (0.0–0.7)	0.0–2.0	-	-
Ocular	Total	136 (2)	0.3 (0.4)	0.2 (0.0–0.6)	0.0–1.8	-	-
	Adolescent	37 (0)	0.4 (0.4)	0.4 (0.0–0.6)	0.0–1.6	1.000	0.19 (−0.03–0.39) ^1^
	Adult	99 (2)	0.3 (0.4)	0.2 (0.0–0.5)	0.0–1.8	-	-
Anxiety/Mood	Total	136 (2)	0.4 (0.4)	0.2 (0.0–0.6)	0.0–1.8	-	-
	Adolescent	37 (0)	0.6 (0.5)	0.4 (0.2–1.0)	0.0–1.8	0.121	0.33 (0.12–0.51) ^1^
	Adult	99 (2)	0.3 (0.4)	0.2 (0.0–0.4)	0.0–1.8	-	-
Migraine	Total	136 (2)	0.1 (0.2)	0.0 (0.0–0.2)	0.0–1.0	-	-
	Adolescent	37 (0)	0.2 (0.3)	0.0 (0.0–0.2)	0.0–1.0	1.000	0.17 (−0.05–0.37) ^1^
	Adult	99 (2)	0.1 (0.2)	0.0 (0.0–0.2)	0.0–0.8	-	-
Vestibular	Total	136 (2)	0.1 (0.3)	0.0 (0.0–0.2)	0.0–1.2	-	-
	Adolescent	37 (0)	0.3 (0.4)	0.2 (0.0–0.4)	0.0–1.2	0.006	0.38 (0.18–0.55) ^1^
	Adult	99 (2)	0.1 (0.2)	0.0 (0.0–0.2)	0.0–1.0	-	-
Sleep	Total	136 (2)	0.4 (0.4)	0.2 (0.0–0.5)	0.0–2.2	-	-
	Adolescent	37 (0)	0.4 (0.5)	0.2 (0.0–0.5)	0.0–2.2	1.000	−0.02 (−0.23–0.20) ^1^
	Adult	99 (2)	0.3 (0.4)	0.2 (0.0–0.5)	0.0–1.5	-	-
Neck	Total	136 (2)	0.2 (0.4)	0.0 (0.0–0.0)	0.0–3.0	-	-
	Adolescent	37 (0)	0.2 (0.4)	0.0 (0.0–0.0)	0.0–1.5	1.000	0.03 (−0.19–0.24) ^1^
	Adult	99 (2)	0.2 (0.4)	0.0 (0.0–0.0)	0.0–3.0	-	-

^1^ = Rank-biserial correlation; m/ = number of participants missing; SD = Standard deviation; IQR = Interquartile range.

**Table 4:** Descriptive statistics for Vestibular–Ocular Motor Screening and Immediate Post-Concussion Assessment and Cognitive Testing outcomes.

Outcome	Age	N (m/)	Mean (SD)	Median (IQR)	Range	*p* -Value	Effect Size
**Vestibular–Ocular Motor Screening**							
Total Score	Total	134 (4)	3.4 (5.4)	1.0 (0.0–4.0)	0.0–28.0	-	-
	Adolescent	37 (0)	3.7 (6.9)	0.0 (0.0–4.0)	0.0–28.0	1.000	−0.13 (−0.34–0.09) ^1^
	Adult	97 (4)	3.2 (4.8)	1.0 (0.0–4.0)	0.0–25.0	-	-
Smooth Pursuits	Total	134 (4)	0.2 (0.5)	0.0 (0.0–0.0)	0.0–2.0	-	-
	Adolescent	37 (0)	0.3 (0.5)	0.0 (0.0–1.0)	0.0–2.0	1.000	0.02 (0.00–0.17) ^2^
	Adult	97 (4)	0.2 (0.4)	0.0 (0.0–0.0)	0.0–2.0	-	-
Horizontal Saccades	Total	134 (4)	0.4 (0.7)	0.0 (0.0–1.0)	0.0–3.0	-	-
	Adolescent	37 (0)	0.3 (0.7)	0.0 (0.0–0.0)	0.0–3.0	1.000	0.03 (0.00–0.20) ^2^
	Adult	97 (4)	0.5 (0.7)	0.0 (0.0–1.0)	0.0–3.0	-	-
Vertical Saccades	Total	134 (4)	0.6 (1.0)	0.0 (0.0–1.0)	0.0–5.0	-	-
	Adolescent	37 (0)	0.5 (0.8)	0.0 (0.0–1.0)	0.0–2.0	1.000	0.02 (0.00–0.16) ^2^
	Adult	97 (4)	0.7 (1.0)	0.0 (0.0–1.0)	0.0–5.0	-	-
Near Point of Convergence	Total	134 (4)	0.4 (1.0)	0.0 (0.0–0.0)	0.0–7.0	-	-
	Adolescent	37 (0)	0.7 (1.6)	0.0 (0.0–1.0)	0.0–7.0	1.000	0.04 (0.00–0.21) ^2^
	Adult	97 (4)	0.3 (0.8)	0.0 (0.0–0.0)	0.0–5.0	-	-
Horizontal Vestibulo-Ocular Reflex	Total	134 (4)	0.6 (1.2)	0.0 (0.0–1.0)	0.0–7.0	-	-
	Adolescent	37 (0)	0.7 (1.6)	0.0 (0.0–1.0)	0.0–7.0	1.000	0.00 (0.00–0.00) ^2^
	Adult	97 (4)	0.5 (1.0)	0.0 (0.0–1.0)	0.0–5.0	-	-
Vertical Vestibulo-Ocular Reflex	Total	134 (4)	0.5 (1.1)	0.0 (0.0–1.0)	0.0–6.0	-	-
	Adolescent	37 (0)	0.7 (1.5)	0.0 (0.0–1.0)	0.0–6.0	1.000	0.03 (0.00–0.19) ^2^
	Adult	97 (4)	0.5 (0.9)	0.0 (0.0–1.0)	0.0–5.0	-	-
Visual Motion Sensitivity	Total	134 (4)	0.6 (1.2)	0.0 (0.0–1.0)	0.0–7.0	-	-
	Adolescent	37 (0)	0.5 (1.3)	0.0 (0.0–0.0)	0.0–6.0	1.000	0.03 (0.00–0.19) ^2^
	Adult	97 (4)	0.6 (1.2)	0.0 (0.0–1.0)	0.0–7.0	-	-
Near Point of Convergence (cm)	Total	134 (4)	0.6 (1.6)	0.0 (0.0–0.0)	0.0–10.2	-	-
	Adolescent	37 (0)	0.8 (1.9)	0.0 (0.0–0.9)	0.0–10.2	1.000	0.02 (0.00–0.17) ^2^
	Adult	97 (4)	0.6 (1.5)	0.0 (0.0–0.0)	0.0–8.1	-	-
**Immediate Post-Concussion Assessment and Cognitive Testing**							
Verbal Memory	Total	134 (4)	86.9 (10.0)	87.5 (81.0–96.0)	56.0–100.0	-	-
	Adolescent	34 (3)	83.8 (10.5)	85.0 (81.0–91.0)	56.0–99.0	1.000	−0.24 (−0.44–0.02) ^1^ -
	Adult	100 (1)	88.0 (9.6)	88.5 (82.0–96.0)	63.0–100.0	-	
Visual Memory	Total	134 (4)	70.9 (12.0)	73.0 (60.0–79.0)	44.0–97.0	-	-
	Adolescent	34 (3)	67.2 (12.0)	68.0 (57.0–76.0)	44.0–91.0	1.000	−0.25 (−0.44–0.03) ^1^ -
	Adult	100 (1)	72.2 (11.8)	74.0 (62.0–81.5)	48.0–97.0	-	
Visual Motor Speed	Total	134 (4)	37.6 (5.9)	37.4 (34.0–41.7)	22.1–52.4	-	-
	Adolescent	34 (3)	33.4 (5.3)	32.7 (29.3–37.2)	22.1–44.3	<0.001	0.09 (−0.27–0.45) ^2^ -
	Adult	100 (1)	39.0 (5.4)	38.6 (35.7–42.6)	24.5–52.4	-	
Reaction Time	Total	134 (4)	0.6 (0.1)	0.6 (0.6–0.7)	0.5–1.3	-	-
	Adolescent	34 (3)	0.7 (0.1)	0.7 (0.6–0.8)	0.6–0.9	<0.001	0.52 (0.33–0.66) ^1^
	Adult	100 (1)	0.6 (0.1)	0.6 (0.6–0.7)	0.5–1.3	-	-
Post-Concussion Symptom Score	Total	138 (0)	11.3 (12.2)	8.0 (3.0–17.0)	0.0–73.0	-	-
	Adolescent	37 (0)	16.5 (18.1)	9.0 (4.0–23.0)	0.0–73.0	1.000	0.19 (−0.03–0.39) ^1^
	Adult	101 (0)	9.4 (8.6)	7.0 (3.0–15.0)	0.0–46.0	-	-

^1^ = Rank-biserial correlation; ^2^ = Glass’ delta; m/ = number of participants missing; IQR = Interquartile range; SD = Standard deviation.

**Table 5:** Descriptive statistics for mental health questionnaires.

Outcome	Age	N (m/)	Mean (SD)	Median (IQR)	Range	*p*-Value	Effect Size	N (%) Meeting Clinical Cut-off
**Patient Health Questionnaire-9**								
	Total	136 (2)	3.5 (3.4)	3.0 (1.0–5.0)	0.0–19.0	-	-	9 (6.6)
	Adolescent	37 (0)	4.7 (4.8)	3.0 (2.0–7.0)	0.0–19.0	1.000	0.14 (−0.07–0.35) ^1^	5 (13.5)
	Adult	99 (2)	3.1 (2.7)	3.0 (1.0–4.0)	0.0–11.0	-	-	4 (4.0)
**Generalised Anxiety Disorder-7**								
	Total	136 (2)	3.2 (4.2)	1.5 (0.0–4.0)	0.0–21.0	-	-	14 (10.3)
	Adolescent	37 (0)	5.5 (5.5)	4.0 (1.0–9.0)	0.0–21.0	0.071	0.35 (0.15–0.53) ^1^	8 (21.6)
	Adult	99 (2)	2.4 (3.3)	1.0 (0.0–3.5)	0.0–15.0	-	-	6 (6.1)
**Pittsburgh Sleep Quality Index**								
Composite Score	Total	135 (3)	5.1 (2.5)	5.0 (3.0–7.0)	0.0–15.0	-	-	76 (56.3)
	Adolescent	37 (0)	5.5 (2.9)	5.0 (3.0–7.0)	1.0–15.0	1.000	0.08 (−0.14–0.29) ^1^	23 (62.2)
	Adult	98 (3)	5.0 (2.4)	5.0 (3.0–7.0)	0.0–11.0	-	-	53 (54.1)
Component 1: Subjective Sleep Quality	Total	136 (2)	1.1 (0.6)	1.0 (1.0–1.0)	0.0–3.0	-	-	-
	Adolescent	37 (0)	1.1 (0.6)	1.0 (1.0–1.0)	0.0–2.0	1.000	0.07 (0.00–0.17) ^2^	-
	Adult	99 (2)	1.1 (0.6)	1.0 (1.0–1.0)	0.0–3.0	-	-	-
Component 2: Sleep Latency	Total	135 (3)	1.3 (1.0)	1.0 (1.0–2.0)	0.0–3.0	-	-	-
	Adolescent	37 (0)	1.6 (1.1)	2.0 (1.0–3.0)	0.0–3.0	1.000	0.24 (0.00–0.38) ^2^	-
	Adult	98 (3)	1.2 (0.9)	1.0 (1.0–2.0)	0.0–3.0	-	-	-
Component 3: Sleep Duration	Total	136 (2)	0.4 (0.6)	0.0 (0.0–1.0)	0.0–2.0	-	-	-
	Adolescent	37 (0)	0.4 (0.6)	0.0 (0.0–1.0)	0.0–2.0	1.000	0.08 (0.00–0.21) ^2^	-
	Adult	99 (2)	0.5 (0.6)	0.0 (0.0–1.0)	0.0–2.0	-	-	-
Component 4: Sleep Efficiency	Total	136 (2)	0.6 (0.7)	0.0 (0.0–1.0)	0.0–3.0	-	-	-
	Adolescent	37 (0)	0.6 (0.8)	0.0 (0.0–1.0)	0.0–3.0	1.000	0.03 (0.00–0.00) ^2^	-
	Adult	99 (2)	0.6 (0.7)	0.0 (0.0–1.0)	0.0–3.0	-	-	-
Component 5: Sleep Disturbance	Total	136 (2)	0.9 (0.5)	1.0 (1.0–1.0)	0.0–3.0	-	-	-
	Adolescent	37 (0)	1.0 (0.7)	1.0 (1.0–1.0)	0.0–3.0	1.000	0.23 (0.00–0.37) ^2^	-
	Adult	99 (2)	0.9 (0.4)	1.0 (1.0–1.0)	0.0–2.0	-	-	-
Component 6: Use of Sleep Medication	Total	136 (2)	0.0 (0.1)	0.0 (0.0–0.0)	0.0–1.0	-	-	-
	Adolescent	37 (0)	0.0 (0.0)	0.0 (0.0–0.0)	0.0–0.0	1.000	0.05 (0.00–0.22) ^2^	-
	Adult	99 (2)	0.0 (0.1)	0.0 (0.0–0.0)	0.0–1.0	-	-	-
Component 7: Daytime Dysfunction	Total	136 (2)	0.7 (0.6)	1.0 (0.0–1.0)	0.0–3.0	-	-	-
	Adolescent	37 (0)	0.8 (0.8)	1.0 (0.0–1.0)	0.0–3.0	1.000	0.15 (0.00–0.29) ^2^	-
	Adult	99 (2)	0.7 (0.6)	1.0 (0.0–1.0)	0.0–2.0	-	-	-
**Neck Bournemouth Questionnaire**								
	Total	136 (2)	6.0 (8.4)	3.0 (0.5–7.5)	0.0–55.0	-	-	-
	Adolescent	37 (0)	7.9 (9.2)	5.0 (1.0–12.0)	0.0–29.0	1.000	0.15 (−0.06–0.36) ^1^	-
	Adult	99 (2)	5.2 (8.0)	3.0 (0.0–6.0)	0.0–55.0	-	-	-
**Migraine Disability Assessment**								
	Total	136 (2)	2.3 (4.9)	0.0 (0.0–2.0)	0.0–27.0	-	-	-
	Adolescent	37 (0)	4.0 (6.8)	0.0 (0.0–6.0)	0.0–25.0	1.000	0.14 (−0.08–0.34) ^1^	-
	Adult	99 (2)	1.6 (3.8)	0.0 (0.0–1.5)	0.0–27.0	-	-	-

^1^ = Rank-biserial correlation; ^2^ = Cramer’s V; m/ = number of participants missing; SD = Standard deviation; IQR = Interquartile range.

**Table 6:** Descriptive statistics for neck dynamometry outcomes.

Neck Dynamometry	Age	N (m/)	Mean (SD)	Median (IQR)	Range	*p* -Value	Effect Size
Neck Flexion (N/kg)	Total	118 (20)	1.2 (0.3)	1.1 (1.0–1.4)	0.4–1.9	-	-
	Adolescent	30 (7)	1.2 (0.4)	1.1 (0.9–1.5)	0.4–1.9	1.000	0.09 (−0.44–0.62) ^1^
	Adult	88 (13)	1.1 (0.3)	1.1 (1.0–1.4)	0.5–1.8	-	-
Neck Extension (N/kg)	Total	118 (20)	1.3 (0.3)	1.3 (1.0–1.5)	0.3–2.2	-	-
	Adolescent	30 (7)	1.3 (0.4)	1.3 (1.0–1.6)	0.3–2.2	1.000	0.13 (−0.35–0.61) ^1^
	Adult	88 (13)	1.3 (0.3)	1.2 (1.0–1.5)	0.6–2.1	-	-
Dominant Side Lateral Flexion (N/kg)	Total	118 (20)	1.0 (0.2)	1.0 (0.9–1.2)	0.4–1.5	-	-
	Adolescent	30 (7)	1.0 (0.2)	1.1 (0.9–1.2)	0.4–1.5	1.000	0.03 (−0.20–0.27) ^2^
	Adult	88 (13)	1.0 (0.2)	1.0 (0.9–1.2)	0.5–1.5	-	-
Non-Dominant Side Lateral Flexion (N/kg)	Total	118 (20)	1.0 (0.2)	1.0 (0.8–1.2)	0.3–1.5	-	-
	Adolescent	30 (7)	0.9 (0.2)	0.9 (0.8–1.1)	0.3–1.4	0.054	−0.29 (−0.71–0.14) ^1^
	Adult	88 (13)	1.0 (0.2)	1.0 (0.8–1.2)	0.4–1.5	-	-
Dominant Side Rotation (N/kg)	Total	118 (20)	0.7 (0.2)	0.7 (0.6–0.8)	0.3–1.2	-	-
	Adolescent	30 (7)	0.7 (0.2)	0.7 (0.5–0.8)	0.3–1.2	0.230	−0.04 (−0.27–0.20) ^2^
	Adult	88 (13)	0.7 (0.2)	0.7 (0.6–0.8)	0.3–1.2	-	-
Non-Dominant Side Rotation (N/kg)	Total	118 (20)	0.7 (0.2)	0.6 (0.5–0.8)	0.2–1.3	-	-
	Adolescent	30 (7)	0.7 (0.2)	0.6 (0.5–0.8)	0.2–1.3	0.339	0.09 (−0.44–0.62) ^1^
	Adult	88 (13)	0.6 (0.2)	0.6 (0.6–0.8)	0.3–1.1	-	-

^1^ = Rank-biserial correlation; ^2^ = Glass’ delta; m/ = number of participants missing; IQR = Interquartile range; SD = Standard deviation.

**Figure A1:**
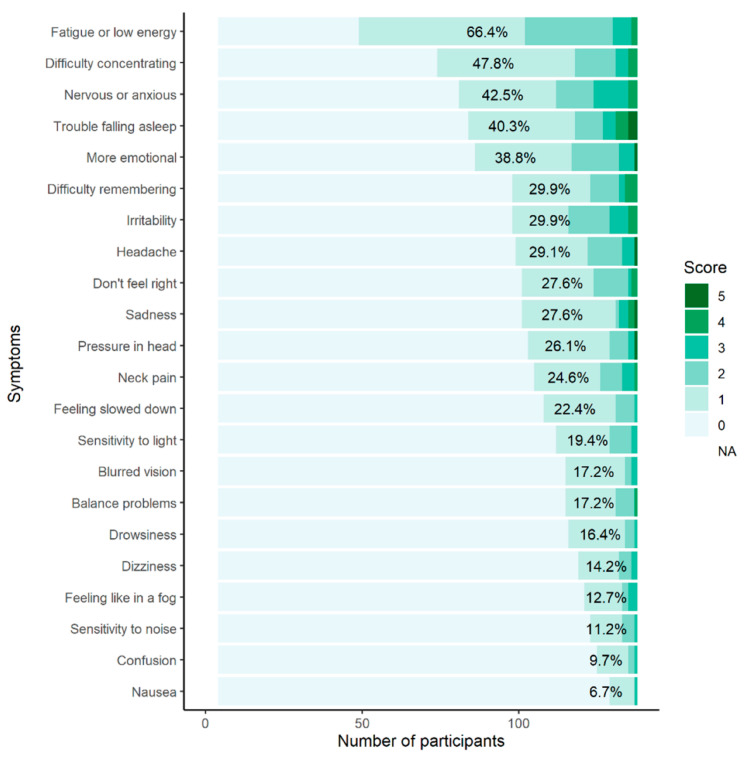
The percentage of participants reporting individual symptoms on the Sport Concussion Assessment Tool 5th Edition Graded Symptom Checklist. The percentages represent participants who scored ≥1 on individual symptoms.

**Figure A2:**
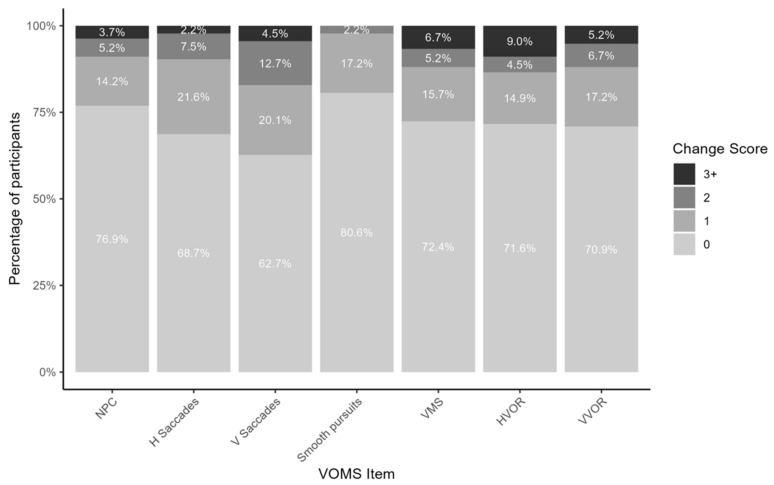
Percentage of participants with change scores of 0, 1, 2, and 3+ on vestibular–ocular motor screening. NPC: near point of convergence; H Saccades: horizontal saccades; V Saccades: vertical saccades; VMS: visual motion sensitivity; HVOR: horizontal vestibular–ocular reflex; VVOR: vertical vestibular–ocular reflex.

## Data Availability

The raw data supporting the conclusions of this article will be made available by the authors on request.

## Appendix A

**Table A1:** Description of all tests included in the multi-domain concussion assessment.

Test	Domain	Description	Scoring and Interpretation	Validity	Reliability
Sport Concussion Assessment Tool 5th Edition [34,35]	Cognitive, vestibular	A composite sideline SRC assessment tool incorporating a graded symptom checklist (GSC), a standardised assessment of concussion (SAC), a neurological examination, and the modified Balance Error Scoring System (mBESS). GSC: Participants reported “how they typically felt” by rating the severity of 22 symptoms on a self-report form. SAC: Participants were tested using a brief, cognitive assessment including orientation questions, immediate and delayed recall of a 10-word list, and a concentration test. Neurological examination: Finger-to-nose and tandem gait tests were carried out. Participants were required to perform five repetitions of a finger-to-nose test as fast as possible using their non-dominant arm. To perform the tandem gait test, participants walked as quickly as possible along a 3-metre line of tape on the floor using tandem gait, turned 180° at the end of the tape, and walked back along the tape to the starting position using tandem gait. mBESS: Balance was assessed by recording the number of standardised errors participants made while performing 20 s holds under three static conditions (double-leg, single-leg, and tandem gate stance).	GSC: A total symptom score (TSS) was calculated by counting the number of symptoms a participant reported (0–22), while a symptom severity score was calculated by summing the severity scores of all symptoms reported (0–132). SAC: Orientation (0–5), immediate recall (0–30), concentration (0–5), and delayed recall (0–10) scores were calculated. Neurological examination: The time in seconds taken to complete the finger-to-nose and tandem gait tests were recorded. If a participant was unable to successfully complete the test, it was recorded as a “fail”. mBESS: The total number of errors for each condition were summed, with a maximum score of ten permitted per condition (0–10). The total number of errors across the three conditions were then summed to form a total score (0–30).	SSS: sensitivity of 73.0%, specificity of 80.0% (AUC = 0.82) within 48 h of SRC [92] SAC: total score has a sensitivity of 61.0% and a specificity of 48.0% (AUC = 0.56) within 48 h of SRC [92] mBESS: Sensitivity of 29.0% and specificity of 85.0% (AUC = 0.58) within 48 h of SRC [92].	SSS: ICC (2,k) = 0.53 SAC: ICC (2,k) = 0.47 mBESS: ICC (2,k) = 0.68 [92]
Immediate Post-Concussion Assessment and Cognitive Testing (version 3.11.1, baseline option) [25]	Cognitive	Computerised neurocognitive test that aims to identify clinically important changes in cognitive function post-SRC. It includes six subtests and the Post-Concussion Symptom Scale (PCSS), a 22-item self-report measure of concussion symptoms.	Four composite scores were produced (verbal memory, visual memory, visual motor processing speed, and reaction time), as well as a total PCSS score (0–22).	Overall sensitivity of 62.5–83.0% (reliable change in at least one score) [93,94] Overall specificity of 55.9–62.5% [94]	ICC (3,1) = 0.47–0.72 for individual composite scores [95]
Vestibular–Ocular Motor Screening [26]	Vestibular, ocular	A brief concussion assessment tool focusing on vestibular–ocular dysfunction. Participants performed seven movements to stimulate different domains of vestibular–ocular function: smooth pursuits, horizontal saccades, vertical saccades, near point of convergence, horizontal vestibular–ocular reflex, vertical vestibular–ocular reflex, and visual motion sensitivity.	Before the assessment and directly following each movement, participants rated four symptoms (headache, dizziness, nausea, and fogginess) on an 11-point numerical rating scale (0–10). Scores across the four symptoms were summed for the pre-test and each test item (0–40). Seven change scores were calculated by subtracting the pre-test score from each item score [26]. These scores were also summed to produce a total symptom score (0–280). NPC distance was measured from the tip of the participant’s nose to the target in cm if she experienced diplopia during the NPC test item. A score of ≥2 on any test item or an NPC distance ≥5 cm is regarded as positive cut-off thresholds.	AUC of 0.64–0.78 for individual items [66]	κ = 0.28–0.51 for individual items [95]
Neck Dynamometry [27,28]	Neck strength	Isometric neck flexion, extension, and dominant and non-dominant side lateral flexion strength were assessed following a HHD protocol described by Kubas [27]. Dominant and non-dominant side rotation (sternocleidomastoid) was assessed using a HHD protocol by Cibulka [28]. The HHD (JTECH Commander Echo Wireless Muscle Testing Starting Kit, JTECH Medical, Salt Lake City, UT, USA) was pre-programmed to perform six tests of three trials and was calibrated to detect minimal forces of 4.4 Newtons. Before commencing the test, we explained it to participants using the following standardised description: “I am going to test your neck strength. I’m going to do that by putting this device against your head in various positions. For each movement, I’ll get you to push against me as hard as you and I’ll resist the movement.” The three trials for each test were performed consecutively. Testing was halted if the participant complained of neck pain.	Mean strength values were calculated for each of the six tests and were normalised to participants’ body mass by dividing force in Newtons by body mass in kilograms (N/kg).	No validity data available for protocol used by Kubas [27]. High correlation with isokinetic dynamometry for neck strength (r = 0.99) [96]	No reliability data available for protocol used by Kubas [27]. ICC = 0.91–0.97 for flexion, extension, and lateral flexion [97].
Concussion Clinical Profiles Screen (CP Screen) [18]	All	Concussion symptom screening tool based on the Clinical Profiles model. It consists of 29 items, which capture symptoms associated with all five of the clinical profiles and two modifiers.	Based on how they currently felt, participants rated each symptom as “none”, “mild”, “moderate”, or “severe”. Responses were assigned scores between zero (“none”) and three (“severe”). Total scores for individual clinical profiles and modifiers were calculated by summing all of a profile’s item scores. Average scores were calculated by finding the mean score per profile (i.e., by summing all of a profile’s item scores and dividing by the total number of items in that profile). An overall total score was calculated by summing the 29 item scores.	AUCs of 0.63–0.93 for individual items [18]	Reliability not reported.
Patient Health Questionnaire-9 [29]	Mood	Nine-item questionnaire based on diagnostic criteria for depression.	Participants rated the frequency with which they experienced nine symptoms over the preceding two weeks: “not at all” = 0, “several days” = 1, “more than half the days = 2, and “nearly every day” = 3. The nine item scores were summed to produce a total score ranging from 0 to 27. A total score of 10 or greater is considered the clinical cut-off threshold for a depressive disorder (sensitivity: 70–88%, specificity: 84–93%) [98]. Total scores are also used to grade depression severity (none: 0–4, mild: 5–9, moderate: 10–14, moderately severe: 15–19, severe: ≥20)	Sensitivity of 88% [29] Specificity of 78% [29]	Reliability not reported in healthy participants.
Generalised Anxiety Disorder-7 [30]	Mood	Seven-item questionnaire based on the diagnostic criteria for generalised anxiety disorder.	Participants reported the frequency of their symptoms over the previous two weeks and were assigned scores based on their responses: “not at all” (score of 0), “several days” (score of 1), “more than half the days” (score of 2), or “nearly every day” (score of 3). The seven item scores were summed, resulting in total scores between 0 and 21. The clinical cut-off score of 10 has a sensitivity of 74% and a specificity of 83% for GAD. Scores can be further classified to describe severity (none: 0–4, mild: 5–9, moderate: 10–14, severe: ≥15).	Sensitivity of ≥80% [99]. Specificity of ≥80% [99].	Reliability not reported in healthy participants.
Neck Bournemouth Questionnaire [33]	Neck	Seven-item questionnaire based on the biopsychosocial model of pain and assessing multiple dimensions of neck pain, including pain intensity, disability, mood, and cognitive-behavioural factors.	Participants rated seven 11-point numerical rating scales ranging from 0 to 10, where 0 indicated no pain or dysfunction, and 10 represented extreme symptoms or an inability to carry out activities. Summing all seven item scores yielded a total score with a possible range of 0 to 70. As the NBQ is used to measure changes in neck pain-related outcomes rather than as a diagnostic tool, cut-off thresholds for classifying patients (i.e., as positive or negative) have not been suggested. However, changes of ≥13 points or ≥36% have been shown to identify patients with improvements in neck pain with high accuracy (100% and 94.4%, respectively).	Sensitivity of 97.8–100.0% for individual subscales in detecting a clinically significant improvement (score ≥ 1) [100]. Specificity of 37.5–66.7% for individual subscales in detecting a clinically significant improvement (score ≥ 1) [100].	Reliability not reported.
Migraine Disability Assessment [32]	Post-traumatic migraine	Five questions that quantify disruption to daily activities caused by headaches.	Five questions asked participants how many days over the previous three months they have experienced major disruption to activities (e.g., missing school or work, being unable to complete household tasks). The total number of days from the five questions were tallied. The total number of days of dysfunction were graded: 0–5 days = grade I (little or no disability), 6–10 days = grade II (mild disability), 11–20 days = grade III (moderate disability), and ≥21 days = grade IV (severe disability).	Items correlated with a migraine diary in migraine patients (ρ = 0.41–0.76) [101].	Overall score: ρ = 0.84 [36]
Pittsburgh Sleep Quality Index [31]	Sleep	The PSQI assesses overall sleep quality. It includes 19 questions that contribute to seven sleep component scores: subjective sleep quality, sleep latency, sleep duration, sleep efficiency, sleep disturbances, use of sleep medication, and daytime dysfunction.	Component scores were calculated according to a complex scoring system and range from 0 to 3. A global PSQI score was computed by adding all seven component scores, giving a range of 0 to 21. A clinical cut-off threshold of 5 has been suggested to identify individuals with poor sleep quality.	Known-group validity for sleep problems in clinical and non-clinical samples (*p* < 0.0001, I^2^ = 93%) [102].	ICC (2,1) = 0.55 [75]

AUC: Area under the curve; ICC (2,k) = Intraclass correlation coefficient two-way random effects model for average measures; ICC (3,1): Intraclass correlation coefficient two-way mixed effects model for single measures; κ: Kappa coefficient; ICC: Intraclass correlation coefficient, model not specified; ρ: Spearman’s rank correlation coefficient; ICC (2,1): Intraclass correlation coefficient two-way random effect model for single measures.
